# GLP-1RAs and cardiovascular disease: is the endothelium a relevant platform?

**DOI:** 10.1007/s00592-023-02124-w

**Published:** 2023-07-04

**Authors:** Rossella Menghini, Viviana Casagrande, Stefano Rizza, Massimo Federici

**Affiliations:** 1 Departments of Systems Medicine, University of Rome “Tor Vergata”, Rome, Italy; 2 Center for Atherosclerosis, Policlinico Tor Vergata, Rome, Italy

**Keywords:** Diabetes, GLP-1 receptor agonists, Endothelium, Atherosclerosis

## Abstract

Hyperglycemia strongly affects endothelial function and activation, which in turn increases the risk of atherosclerotic cardiovascular disease. Among pharmacotherapies aimed at lowering blood glucose levels, glucagon-like peptide 1 receptor agonists (GLP-1RA) represent a class of drugs involved in the improvement of the endothelium damage and the progression of cardiovascular diseases. They show antihypertensive and antiatherosclerotic actions due at least in part to direct favorable actions on the coronary vascular endothelium, such as oxidative stress reduction and nitric oxide increase. However, cumulative peripheral indirect actions could also contribute to the antiatherosclerotic functions of GLP-1/GLP-1R agonists, including metabolism and gut microbiome regulation. Therefore, further research is necessary to clarify the specific role of this drug class in the management of cardiovascular disease and to identify specific cellular targets involved in the protective signal transduction. In the present review, we provide an overview of the effects of GLP-1RAs treatment on cardiovascular disease with particular attention on potential molecular mechanisms involving endothelium function on formation and progression of atherosclerotic plaque.

## Introduction

The intuition that endothelial damage could contribute to the development of vascular pathologies causing an increase in morbidity and mortality dates back to many years ago; however, the study of the mechanisms underlying this process is much more recent and constantly evolving [1]. The endothelial cell (EC) plays many important functions throughout the body and shows vascular-specific heterogeneity [2]. It is now clear that the endothelium, in addition to the barrier function and regulation of cell permeability, has autocrine, paracrine and endocrine functions, controlling blood flow and pressure, hemostasis and coagulation, immune and inflammatory responses, vasculogenesis and angiogenesis [3]. The functional alteration of the endothelium is closely linked to a reduction in nitric oxide (NO) bioavailability, but also to the modulation of platelet markers, proinflammatory factors and adhesion molecules. Therefore, in addition to functional measures of endothelial dysfunction, molecules including tumor necrosis factor alpha (TNF-α), intercellular adhesion molecule 1 (ICAM-1) and vascular cell adhesion molecule 1 (VCAM-1) can also be considered surrogate markers of endothelial dysfunction and cardiovascular risk [4].

Cardiovascular risk factors, such as hyperglycemia, hypercholesterolemia, hypertension, smoking and oxidative stress, are crucial mediators of endothelial dysfunction, defined as the decrease in synthesis, release and activity of the endothelium derived NO, while proinflammatory cytokines, increased flow and advanced glycation endproducts (AGEs) are classical mediators of EC activation, defined by increased endothelial expression of cell surface adhesion molecules [5]. However, the reduction of NO leads to increased endothelial activation and, in parallel, the activation of ECs prompts endothelial dysfunction. Moreover, both endothelial dysfunction and activation lead to atherosclerosis and vascular disease by increasing vasoconstriction, smooth muscle cells (SMC) proliferation, platelet aggregation, leukocyte adhesion, LDL oxidation and matrix metalloproteinases (MMP) activation [6].

Prolonged hyperglycemia as well as transient acute hyperglycemia impairs endothelial function. Therefore, pharmacotherapies aimed at reducing blood glucose levels are supposed to be able to reduce the endothelium damage and the progression of cardiovascular diseases. However, not all agents used to treat diabetes have been shown to reduce the risk of cardiovascular disease, despite the effective lowering of blood glucose [7]. Intestinal L cell-derived GLP-1 is a postprandial peptide that plays a critical role in the control of blood glucose level by increasing insulin secretion in pancreatic β cells and suppressing glucagon release [8]. Endogenous GLP-1 has a very short elimination half-life of < 1.5 min after intravenous administration due to rapid degradation by the enzyme dipeptidyl peptidase (DPP4) [9]. This evidence has driven the development of DPP4-resistant GLP-1 analogs. The GLP-1RAs currently available can be broadly classified as analogues of human GLP-1 with various structural modifications that prolong its half-life, such as modifications of amino acids (which confer resistance to the action of DPP4) or the addition of a chain of fatty acids (liraglutide), albumin (albiglutide) or immunoglobulin (dulaglutide). Alternatively, there are exendin-based therapies being exendin-4 a synthetic GLP-1RA that shares 53% sequence homology with native GLP-1 [10]. GLP-1RA clinical trials with cardiovascular outcome (CVOT) reported significant reductions in major cardiovascular adverse events (MACE) compared to placebo in five out of seven CVOTs, even regardless of their ability to control blood glucose [11]. However, the main mechanism remains enigmatic. Based on the results of recent CV outcome studies, GLP-1RA drugs compared with sodium–glucose cotransporter 2 (SGLT2) inhibitors have shown, in addition to some common favorable CV effects, several class-specific effects (GLP-1RA: benefits for the risk of atherosclerotic outcomes, inhibitors SGLT2: benefits for the risk of heart failure outcomes) contributing to the overall improvement of CV. The most evident reduction appears to be associated with atherosclerotic pathways suggesting anti-atherogenic properties of GLP-1RA [12, 13]. How this effect is due to a direct vascular action or to the metabolic improvement or whether a protection of the endothelial function is involved remains to be clarified.

## Role of GLP-1RAs in ECs: direct or indirect effects?

Type 2 diabetes (T2D) patients treated with subcutaneous exenatide show that it exerts a protective GLP-1R-dependent effect on postprandial endothelial function, compared to placebo, suggesting direct action on the endothelium [14]. Indeed, there are at least four evidences suggesting the hypothesis that endothelium may represent a direct target of GLP-1RAs: 1. GLP-1R acts in many pathways involved in the regulation of endothelial function [15]; 2. GLP-1R is expressed in several cells involved in the pathogenesis of atherosclerosis, including endothelium and macrophages [16, 17]; 3. The EC represents a direct GLP-1 target [18]; 4. In several in vitro studies, the GLP-1RA treatment exerts protective action in ECs. In fact, GLP-1RAs exert protective effects on the endothelium vascular tone and inflammation, by inducing the activation of nitric oxide synthase, endothelial (eNOS), and NO production in ECs in vitro through the protein kinase AMP-activated catalytic subunit alpha (AMPK)/Akt pathway [14] and suppressing the expression of proinflammatory cytokines, chemokines and adhesion molecules induced by hyperglycemia, inflammatory stimuli or oxidative stress [19–21]. Moreover, GLP-1RAs show anti-adhesive properties by reducing the adhesion of monocytes to ECs at least in part through the regulation of Kruppel-like factor 2 (KLF2), a transcription factor that plays an important protective role in regulating the inflammatory response during atherosclerosis and other cardiovascular diseases [21, 22]. Both in ECs and endothelial progenitor cells (EPCs), GLP-1RA treatment is able to increase SirT6 expression, thus reducing inflammatory pathways induced by high glucose. The relevance of the obtained results is confirmed by the evidence that compared with non-GLP-1 therapy-treated plaques, GLP-1 therapy-treated plaques presented greater SIRT6 expression and collagen content, and less inflammation and oxidative stress, indicating a more stable plaque phenotype [23]. Furthermore, in ECs, incretin treatment has been shown to be involved in the regulation of adiponectin/APPL1 signaling pathway, thus preventing atherosclerosis progression or plaque vulnerability in type 2 diabetes patients [24]. The trans-differentiation of ECs into mesenchymal cells (EndMT) plays a vital role in cardiovascular diseases, including atherosclerosis [25]. Recent studies have revealed that inflammation and diabetic conditions trigger EndMT [26, 27]. GLP-1RAs inhibits EndMT markers expression induced by glucose or IL-1b in ECs via regulation of AMPK [28]. Epigenetics mechanisms, through DNA methylation, histone modifications and non-coding RNA regulation, have been shown to play a key role in the pathophysiological mechanism of diabetes and progression of CVD [29]. Recent data indicate that in ECs, treatment with GLP-1 agonists prevented high glucose-induced demethylation in the promoter region of nuclear factor-kB (NF-kB) and superoxide dismutase 2 (SOD2), avoiding their detrimental expression, suggesting a potential role of GLP-1 and GLP-1R agonists on epigenetic machinery to prevent the vascular diabetic complications. Accordingly, diabetic patients showed a decrease in DNA methylation of these genes compared to non-diabetic patients [30]. In summary, available evidence suggests a role for GLP-1RAs in reversing endothelial dysfunction; however, it should be considered that in vitro treatments are performed with higher GLP-1RAs dose compared to physiological levels of GLP-1 observed after nutrient ingestion indicating that the reported results could potentially be artifacts due to promiscuous actions on related receptors. Therefore, further evidence is needed to confirm the in vitro observations.

## GLP-1RAs and atherosclerosis: which is the target?

Ex vivo studies using isolated aortic rings obtained from high-fat diet ApoE-/- mice treated with liraglutide showed significant improvement in endothelial function, increased eNOS expression and reduction of ICAM-1, indicating that these drugs may reverse endothelial dysfunction in GLP-R-dependent manner [20]. To facilitate the analysis of systemic independent effects and focus on the direct role of GLP-1RAs in atherosclerotic cells, GLP-1RA doses should be specifically designed to have minimal impact on weight and metabolism. In both, ApoE-/- mice and LDLr-/- mice models of atherosclerosis, liraglutide treatment significantly attenuated atherosclerotic plaque development partly independently of body weight and cholesterol reduction, and transcriptomic analysis showed a reduction of proinflammatory pathways in aortic atherosclerotic tissue, suggesting a direct action on plaque independently of metabolic effects [31].

Other data, obtained from ApoE-/- mice, suggest that exendin-4 could have beneficial effects against atherosclerosis without affecting metabolism and could potentially prevent the progression of atherosclerosis by its direct action on cells involved in atherosclerosis. The data suggest that exendin-4 markedly reduced monocyte/macrophage accumulation in the vascular wall at least in part by suppressing the inflammatory response in macrophages through activation of the cAMP/PKA pathway [32]. Therefore, in addition to the effect of GLP-1RAs on ECs, their effect on monocytes/macrophages may also have a strong impact on the attenuation of atherosclerosis. Moreover, in ApoE-/- mice low liraglutide dose promotes an MΦ2 macrophage phenotype. These changes in the macrophage phenotype reduce deleterious changes in the vascular wall and promote favorable stabilization of atherosclerotic lesions [33]. This observation further suggests the ability of GLP-1RAs to modulate harmful immune responses that drive plaque formation.

Recently, a novel plaque-targeted nano-GLP-1RA (GlpNP) drug has been designed, synthesized and injected at very low doses twice a week for six weeks in ApoE-/- mice to allow decoupling of systemic effects in effort to understand the impact of direct plaque delivery on atherosclerosis. Collectively, the obtained data indicated that GlpNP can selectively deliver into atherosclerotic plaque, where it appears to accumulate in vascular smooth muscle cell (VSMC)-like cells. Moreover, it favorably modulates atherosclerosis with pancreatic or central nervous system-independent effects and without significant changes in metabolic parameters, suggesting a direct action of GLP-1 analogues on atherosclerosis, involving cholesterol efflux and inflammation, especially in the context of SMC inflammation [34]. In mice with experimental arterial hypertension, liraglutide reduced the angiotensin II-induced inflammatory cascade and oxidative stress in the vascular wall and thus restoring NO bioavailability and protecting from angiotensin II-induced endothelial dysfunction. Interestingly, the vascular protective effects were abolished in global Glp1r-/- mice and in mice with selective disruption of GLP-1R expression in EC but not in the myeloid lineage, indicating that endothelial GLP-1R mediates cardiovascular protection by the GLP-1RA liraglutide-independent glycemic control [35]. In summary, these findings indicate that GLP-1RAs have, at least in part, a direct effect on cardiovascular protection through the involvement of multiple pathways in different cell types.

## GLP-1R dependent or independent pathways in cardiovascular disease: this is the question

As already mentioned, the native GLP-1, 7-36a, has a short half-life (1–2 min) due to rapid enzymatic degradation by DPP4 and other enzymes [36]. The form of GLP-1, 9-36a, cleaved with DPP4, is the predominant circulating form [37]. It was previously perceived as an inactive GLP-1 derivative, due to the evidence that 9-36a did not show any significant insulin-stimulating effects and a weak affinity for the GLP-1R [38]. However, several studies demonstrated that 9-36a may have direct effects on the cardiovascular system [39]. Accumulating evidence from in vivo, ex vivo and in vitro studies indicates that GLP-1(7–36) amide degradation products may exert their own CV protective effects independent of those known to affect the GLP-1R [17]. Both native GLP-1 and its metabolite GLP-1(9–36) amide have a vasodilatory action and still induce vasodilation in arteries from GLP-1R knockout mice, which strongly support the evidence of a vasodilator signaling mechanism not mediated by canonical GLP-1R [16]. GLP-1(9–36) amide enhanced human aortic EC viability in response to hypoxia injury and hydrogen peroxide treatment via a NO and mitochondrial-dependent mechanism [40]. Moreover, the evidence that native GLP-1, as well as its metabolites, had comparable anti-atherosclerotic effects implies that this cardiovascular benefit is mediated by both GLP-1R dependent and independent pathways [41]. This evidence may explain the effects on cells and tissues in which expression of a functional GLP-1R is questionable; on the other hand, it raises the question of whether there is a second, probably yet unexplored GLP-1R. However, the relevance of GLP-1 degradation products to the beneficial effects of GLP-1RAs in clinical trials is questionable and needs further research as they are only minimally degradable.

## Endothelial progenitor cells: new players involved in GLP-1RAs-dependent cardiovascular protection?

EPCs are bone marrow-derived or tissue-resident cells that play a vital role in maintaining vascular integrity and repairing endothelial damage [42]. Systemic oxidative stress and inflammation alter the potential of vascular regenerative cells in the bone marrow by impairing blood vessel repair [43]; a reduction in the number of circulating EPCs has been linked to an increased risk of cardiovascular disease [44]. Recently, the investigation of possible direct and indirect effects of GLP-1R activation on the differentiation and maturation of vascular progenitor cells demonstrated that, in EPCs stimulated with high glucose, GLP-1R expression is reduced. GLP-1R knockdown is associated with increased EPC apoptosis and reduced EPC migration, adhesion and angiogenicity capabilities [45]. Additionally, treatment with exendin-4 improved EPCs function after high-glucose stimulation [46]. Thus, GLP-1R expression appears to play important roles in regulating EPC dysfunction in hyperglycemia.

## GLP-1RA effect on endothelial metabolism: a new avenue to explore in cardiovascular disease

**Fig. 1:**
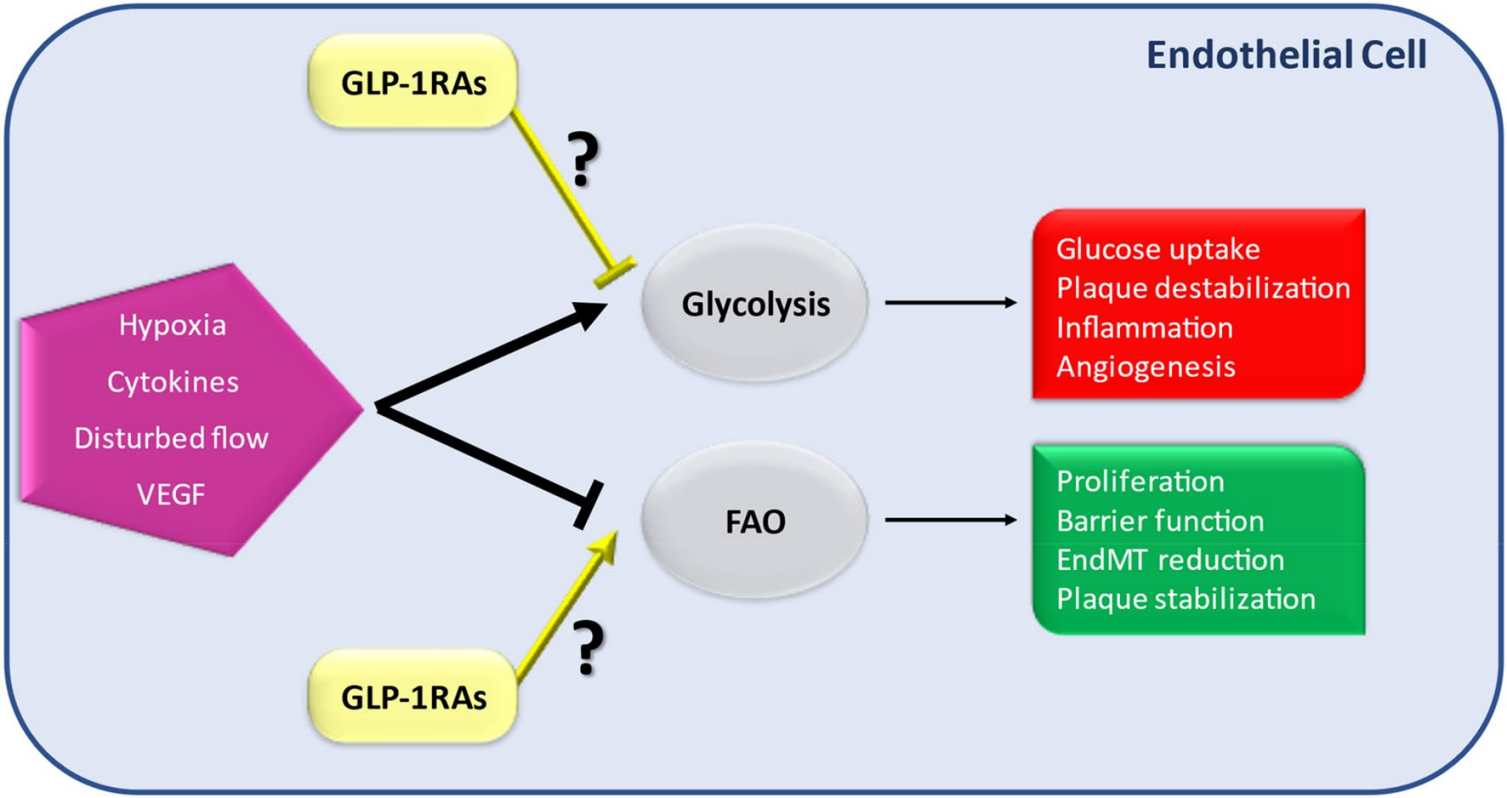
Schematic diagram illustrating the hypothesis for GLP-1RAs effects on endothelial metabolism

Metabolic pathways have emerged as key regulators of many EC functions, including angiogenesis, inflammation and barrier function, processes that are deregulated during atherogenesis [47]. In particular, limiting glycolysis or stimulating FAO in ECs may represent a therapeutic strategy against atherosclerosis [48]. In fact, proinflammatory cytokines increase glucose uptake and glycolysis in ECs leading to NF-κB activation, whereas fatty acid oxidation (FAO maintains) is involved in reduction of FA-induced EC dysfunction and apoptosis [49, 50], in the protection of EC barrier function [51] and in the EndMT inhibition [52], suggesting that endothelial FAO may reduce atherosclerosis development. Interestingly, several studies in other cells indicate that GLP-1 might be a good candidate able to induce fatty acid oxidation at the expense of reduced glucose utilization (Fig. 1).

GLP-1 inhibits glucose uptake and promotes β-oxidation in cultured astrocytes [53]. GLP-1RAs treatment reduces adiposity by promoting lipolysis, fatty acid oxidation and mitochondrial biogenesis in the WAT, liver, muscle and BAT of obese mice and in 3T3-L1 adipocytes and shows amelioration of liver steatosis by promoting mitochondrial fatty acid β-oxidation and inhibiting lipogenesis in vivo and in vitro [54, 55]. Moreover, exposure to GLP-1 increases energy expenditure in muscle at least in part through the upregulation of fat oxidation [56]. Recently, disruption of the gut microbiota has been associated with a reduction of eNOS activity in cerebral ECs highlighting the potential of the microbiota as a target to reverse endothelial dysfunction [57]. There is evidence that GLP-1 may play a role in the function of the intestinal epithelium, correlating these effects with changes in gut microbiota. In a recent work, we have found that a fixed combination of insulin degludec and liraglutide ameliorates quality of life and depression [58] significantly impacting on both gastrointestinal microbes and cognitive function in elderly T2D individuals [59]. In particular, our results revealed a significant increase in gut Gram-negative Alistipes content associated directly with cognitive improvement and inversely with TNF-α levels suggesting that the entanglement between gut microbiomes modulation and neuro-inflammation needs to be further investigated.

## Conclusions

GLP-1RAs are a new class of drugs used for the treatment of T2DM. They are not only able to improve the hyperglycemia in diabetic patients but also to modulate other significant risk factors for CVD such as high blood pressure, dyslipidemia or obesity. These properties explain, at least in part, the positive results of CV outcome studies. GLP-1RAs appear to elicit CV protection both directly in the vasculature and indirectly in the periphery (Table 1).

**Table 1:** A summary of the major mechanisms of action of the GLP-1RAs in cardiovascular disease and metabolic pathways

Model	GLP-1RA	Effects	Ref
*Direct or indirect effects in ECs*			
ECs	Exenatide	eNOS activation	14
ECs	Liraglutide Dulaglutide	Reduced inflammatory markers and adhesion molecules	19–24
ECs	Liraglutide	Reduced monocytes adhesion to endothelial cells	22
ECs	Liraglutide	Inhibition of EndMT markers	28
ECs	Liraglutide	Epigenetic regulation	30
*GLP-1RAs and atherosclerosis*			
ApoE-/- mice	Liraglutide	Improvement of endothelial dysfunction	20
ApoE-/- mice and LDLr-/- mice	Liraglutide	Reduced inflammation pathway in atherosclerotic plaques	31
ApoE-/- mice	Exendin-4	Reduced inflammatory response in macrophages	32
ApoE-/- mice	Liraglutide	Induced MΦ2 macrophage phenotype	33
ApoE-/- mice	plaque-targeted nano-GLP-1RA	Reduced atherosclerosis and VSMC inflammation	34
Experimental arterial hypertension	Liraglutide	Endothelial GLP-1R mediates cardiovascular protection	35
*GLP-1R dependent or independent pathways*			
Glp1r − / − mice	GLP-1(9–36)	Induced vasodilation in arteries	16
ECs	GLP-1(9–36)	Protection from ischemia reperfusion injury	40
ApoE-/- mice	GLP-1(9–37) and GLP-1(28–37)	Stabilization of atherosclerotic lesions	41
*Endothelial progenitor cells*			
EPCs		GLP-1R expression is reduced with HG	45
EPCs	Exendin-4	Improved cell functions during HG treatment	46
*Metabolism*			
Astrocytes	GLP-1	glucose uptake inhibition and promotion of β-oxidation	53
HFD mice	Exenatide	Promotion of fatty acid oxidation and mitochondrial biogenesis	54
HFD mice	Liraglutide	Enhanced fatty acid β-oxidation	55
Elderly T2D individuals	Insulin Degludec and Liraglutide	Cognitive improvement and increase in gut Gram-negative Alistipes	59

**Fig. 2:**
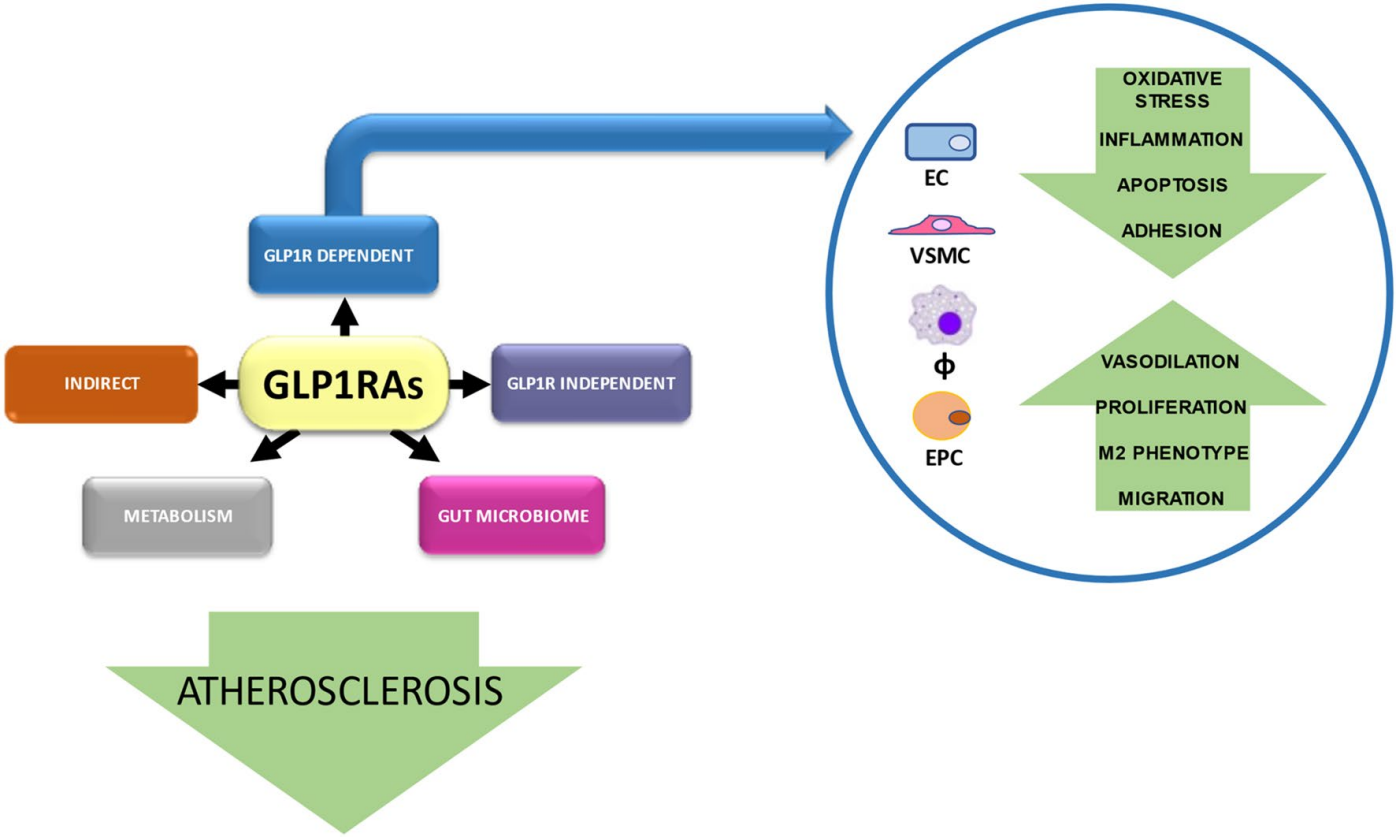
Potential actions of GLP-1RAs in improving vascular function and atherosclerosis. GLP-1RAs mediate a number of favorable actions, which support the protective role during atherosclerosis

However, what is not clear is the proportion of this CV protective mechanism that is driven by local GLP-1R stimulation versus the cumulative peripheral effects that may indirectly improve vascular function and atherosclerosis (Fig. 2). Furthermore, although the GLP-1R-dependent effects of these compounds are at least partially characterized, the presence of any GLP-1R-independent, pleiotropic effects on the CV system requires much more investigation, particularly regarding their signal pathways.
